# Assessing connectivity and the contribution of private lands to protected area networks in the United States

**DOI:** 10.1371/journal.pone.0228946

**Published:** 2020-03-05

**Authors:** Lindsey Bargelt, Marie-Josée Fortin, Dennis L. Murray

**Affiliations:** 1 Department of Biology, Trent University, Peterborough, ON, Canada; 2 Department of Ecology and Evolutionary Biology, University of Toronto, ON, Canada; 3 Department of Biology, Trent University, Peterborough, ON, Canada; Institute of Geographic Sciences and Natural Resources Research Chinese Academy of Sciences, CHINA

## Abstract

Current targets for protected area network coverage call for increased protection but lack specificity in terms of criteria for parcel type, placement, and landscape connectivity. We assessed land conservation achieved by protected area networks in the contiguous United States, and assessed whether private lands contributed substantially to network coverage and connectivity given species dispersal abilities. On average, states have 4.1% (range: 0.2% to 15.8%, *n* = 48) protected area coverage with connectivity ≤10 km. Terrain ruggedness, percent farmland, and population density are the primary determinants of protected area placement, leading to biased representation of land features currently under protection. On average, private protected areas contribute <1.1% (range 0.0% to 9.2%) to current protected area coverage across the United States. We conclude that current levels of protection and connectivity are inadequate to support a functional protected area network, and that increased and strategic selection of private protected areas is necessary to meet conservation planning goals.

## Introduction

Protected areas are a longstanding cornerstone in conservation biology, serving as key components in maintaining and restoring structure and function of natural landscapes and ecosystems [1]. The contribution of protected areas in reaching conservation planning goals is largely a function of biodiversity and habitat quality within individual sites, as well as their physical attributes like size, shape, and proximity to other protected areas [2–4]. Establishment of new protected areas is a priority, as the United Nations Convention on Biodiversity set a global target of protecting 17% of terrestrial land by 2020 [5]. In fact, this target may be too low to forestall major losses in biodiversity, and higher targets recently have been proposed [6,7]. Currently, only 13% of the United States (US) is protected by areas > 1,000 ha [8], even though roughly 45% of the country’s contiguous land mass remains in a natural, albeit fragmented, state [9]. It follows that selection of land parcels for protection should be strategic and prioritize not only areas that retain ecological and evolutionary processes [10], but also those reinforcing contributions of individual sites to larger protected area networks (PANs) [11]. However, to date these criteria have not been fully integrated into conservation planning, and there remains a need to more fully evaluate the selection process for protected areas and their larger contribution to PAN design.

Establishment and design of PANs often is a loosely-structured process, with longstanding efforts to balance the number, size, and proximity of individual sites that are under public ownership and management [12,13]. Although connectivity is usually informally prioritized during the PAN design process [10], proximity of sites may be an important constraint given the limited availability of candidate sites and conflicting demands for land use. However, strategic selection of new protected areas that fill specific gaps in PANs critically promotes dispersal success and gene flow [14], and is anticipatory for processes such as species range loss and distribution shifts [15,16]. Recently, [11] showed that at a national scale the US has major gaps in PAN connectivity, in part because considerable landcover that could otherwise improve landscape connectivity is privately-owned and thus, traditionally, not available [17]. There is a recent effort to integrate private areas in PANs if such areas meet conservation needs and can be adequately protected through legal frameworks. About 57% of naturally-connected land in the US is privately-owned [17], but, the potential value of turning private land into protected areas to conservation planning remains largely unclear [18,19].

Gaps in PANs may also arise through further constraints placed on the availability of candidate sites. In the US, individual protected areas historically were selected for scenic rather than conservation value [20], and low agricultural opportunity [21], resulting in a bias toward sites with low soil productivity and high elevation [22,23]. Accordingly, a variety of ecotypes are under-represented in existing PANs, including arable or moderately habitable land; such landcover may be suitable for improving network functionality, including through improvements to PAN connectivity. Indeed, if strategic protected area growth from 2004–2014 had targeted threatened vertebrates, more than 30 times more species would have been protected for the same area of actual protected area expansion [21].

This paper evaluates connectivity in PANs across the contiguous US, to determine the relative contribution of public and private protected areas, and the corresponding role of socio-economic and geographic factors, on PAN connectivity. We identified gaps in network connectivity across the 48 contiguous states and predicted that: (1) states with more or larger protected areas would have higher structural connectivity across PANs, and that (2) private protected areas would contribute nominally to PAN connectivity. Because establishment of PANs could be influenced by a range of socio-economic and geographical factors, we also predicted that: (3) terrain ruggedness [topographic heterogeneity, see Riley et al. [24] and percent farmland would be the primary factors affecting protected area coverage and connectivity, with influence from regional population density and/or per capita income [25,26]. Our quantitative assessment of PAN connectivity and the contribution of private protected areas provides insights into the role of private lands in conservation planning.

## Methods

### Structural connectivity of protected areas

We determined landscape protection and connectivity for each state in the contiguous US by using connectivity indices based on network theory [27,28]. This approach provides a measure of structural connectivity that can be applied to large landscapes without the data intensity required for functional connectivity analysis (i.e., resistance mapping) [29,30]. For each state, we calculated a protected-connected metric “ProtConn” that quantified landscape connectivity between protected areas relative to potential dispersal distances between them [28], allowing us to encompass both the broad spatial scale of our study region and variable dispersal distances of a range of species. This network approach serves as basis for implementing Aichi Target 11 of the Convention on Biological Diversity [5,28,11]. By treating each state as a distinct unit with a discrete network of protected areas (as many are a result of state policy and designated by local entities), we obtained replicate samples to assess how landscape and anthropogenic metrics relate to protection and connectivity; our approach is not designed to measure PANs spanning multiple states and thus is not a measure of inter-state connectivity.

For each state, we compiled spatial information for public and private protected areas using two datasets: National Conservation Easement Database (NCED) (https://www.conservationeasement.us/, accessed August 2017) and the public version of the World Database on Protected Areas (WDPA) (Protected Planet (http://www.protectedplanet.net/, accessed August 2017)). The NCED is developed by the U.S. Endowment for Forestry and Communities, and contains spatial information, ownership, and holder information for conservation easements (i.e., private protected areas); all easements in the database (*n* = 135,614) were used in our analysis. The NCED contains ~ 49% of all publicly-held easements (i.e., those held by federal, state, regional and local governments), and ~ 75% of easements held by non-profit groups (i.e., land trusts and conservation NGOs) [31]. The WDPA United States dataset is provided by the U.S. Geological Survey’s Gap Analysis Program and includes all national and state-designated protected areas. Thus, given the breadth of organizations submitting data to NCED and WDPA, and the high percentage of reported areas, we inferred that our analysis was not likely to be compromised by reporting bias (see Saura et al. [28]).

Using ArcMap 10.5 [32], we projected NCED and WDPA datasets to the USA Contiguous Albers Equal Area Conic projection and clipped them to each state extracted from a 500k resolution cartographic boundary shapefile [33]. Marine protected areas, and terrestrially protected areas with “proposed” or “not reported” status were excluded, as well as protected areas reported as points with unknown boundaries. Although previous studies drew buffers to represent reported area for points, many locations were duplicates of polygons and the total number of these points was < 1% of the dataset [4,28]. Ultimately, the two datasets yielded 167,997 (99.6% of datasets) polygons of protected areas and conservation easements.

We considered areas listing easement holder type (NCED) or managing authority (WDPA) as ‘NGO’ or ‘private’, as private protected areas [34]. Of all protected areas, 5.1% had ‘unknown’ or ‘not reported’ holder types or managing authority, but these were primarily concentrated in select states (i.e., Missouri and Mississippi). If easement holder name was given in NCED or information on designation or governance type in WDPA, we determined if the site was privately owned, with remaining areas considered public.

To compare privately-funded and public conservation areas, we merged data to form layers for: (i) total protected areas; (ii) public protected areas; and (iii) private protected areas. We then dissolved each layer to remove overlaps between different designations within WDPA and overlaps between NCED and WDPA. As this process combined all overlapping and adjacent polygons, final polygons potentially contained multiple designated protected areas (hereafter a ‘patch’), but eliminated the issue of duplicated polygons. We calculated patch area using the set projection and removed all patches < 1 ha because the dissolving process leaves incongruous slivers. While previous studies exploring similar data have removed patches < 1 km^2^ [28,35], our lower threshold was appropriate given the size of privately-owned land parcels and small spatial scale of landscape protection in many areas [36]. Small habitat patches match the home ranges of many species of conservation concern [37], and have increasingly been found to be of high importance to conserving biodiversity [38,39]. Note that this approach also presents a more accurate measure of patch size and connectivity. We used the ArcGIS Conefor Inputs plugin (http://www.conefor.org/gisextensions.html) to obtain edge-to-edge Euclidian distances between patches, and then the software Conefor 2.6 [40] to calculate the Equivalent Connected Area (ECA). ECA represents the necessary area occupied by a single patch to provide the same area of reachable protected land as the PAN. We then estimated ProtConn for seven dispersal distances (0.5, 1.0, 5.0, 10.0, 30.0, 50.0 and 100.0 km). ProtConn is determined by percent of ECA to maximum landscape attribute, which is the potential coverable patch area [28]. We set this as the projected area (ha) of each state and estimated ProtConn for all patches and public-only patches.

We averaged the difference between ProtConn_All_ (all public and private protected areas) and ProtConn_Public_ (excludes private protected areas) across all dispersal distances for each state, to assess the difference in ProtConn when private protected areas were excluded. To identify connectivity gaps in state PANs, we standardized the difference between percent of total land protected and ProtConn_All._ Herein, we report on dispersal distance (*d*) = 10.0 km; ancillary analyses (see S2 and S4 Tables) show that our results expectedly vary with thresholds (corresponding to species dispersal abilities), although patterns remain qualitatively similar.

### Model selection

We used model selection [41] to assess relationships between landscape protection and connectivity and environmental or anthropogenic factors. We considered 15 multi-variable models developed *a priori* and based on our understanding of potential drivers of connectivity and protected area designations. Seven predictor variables were included for each state: percent farmland; population density; average terrain ruggedness [24]; mean per capita income; political voting pattern (as measured by the last 10 presidential elections (www.270towin.com); median land trust age [42]; and percent of the state covered by private protected areas (see S3 Table for additional details on predictor selection and coding). We considered that, collectively, these predictors potentially influence funding, support, and development of protected areas and thus may explain observed patterns in protected area coverage. Predictors were chosen from an initial set of 10, which excluded those having high collinearity (*r* > 0.70, *n* = 8) (S12 Fig). Using the *glmmADMB* and *bblme* packages in R (version 3.4.3), we used Akaike’s Information Criterion corrected for small sample sizes (AICc) to rank models, and McFadden’s *R*^*2*^ [43] to report model goodness-of-fit. Models with ΔAICc < 2.0 were considered indistinguishable [41].

## Results

### Protected area networks

On average, protected areas (public and private) cover 8.4% of states (range: 0.7% (Kansas), 24.1% (California; S1 Fig)). Regional differences are prominent, with six mostly midwestern states having < 3.0% protection and 11 western states having > 5.5% protection. Coverage specifically provided by private protected areas averaged 1.1% (range: 0.0% (Nevada) to 9.2% (Maine)) (S2 Fig), but potential coverage varied according to federal land management by state. Notably, 46.4% of land in 11 western states is federally managed compared to 4.2% across remaining states; Nevada has 76.9% of its area managed by the federal government, leaving limited space for private protected areas.

### Protected area connectivity

**Fig 1:**
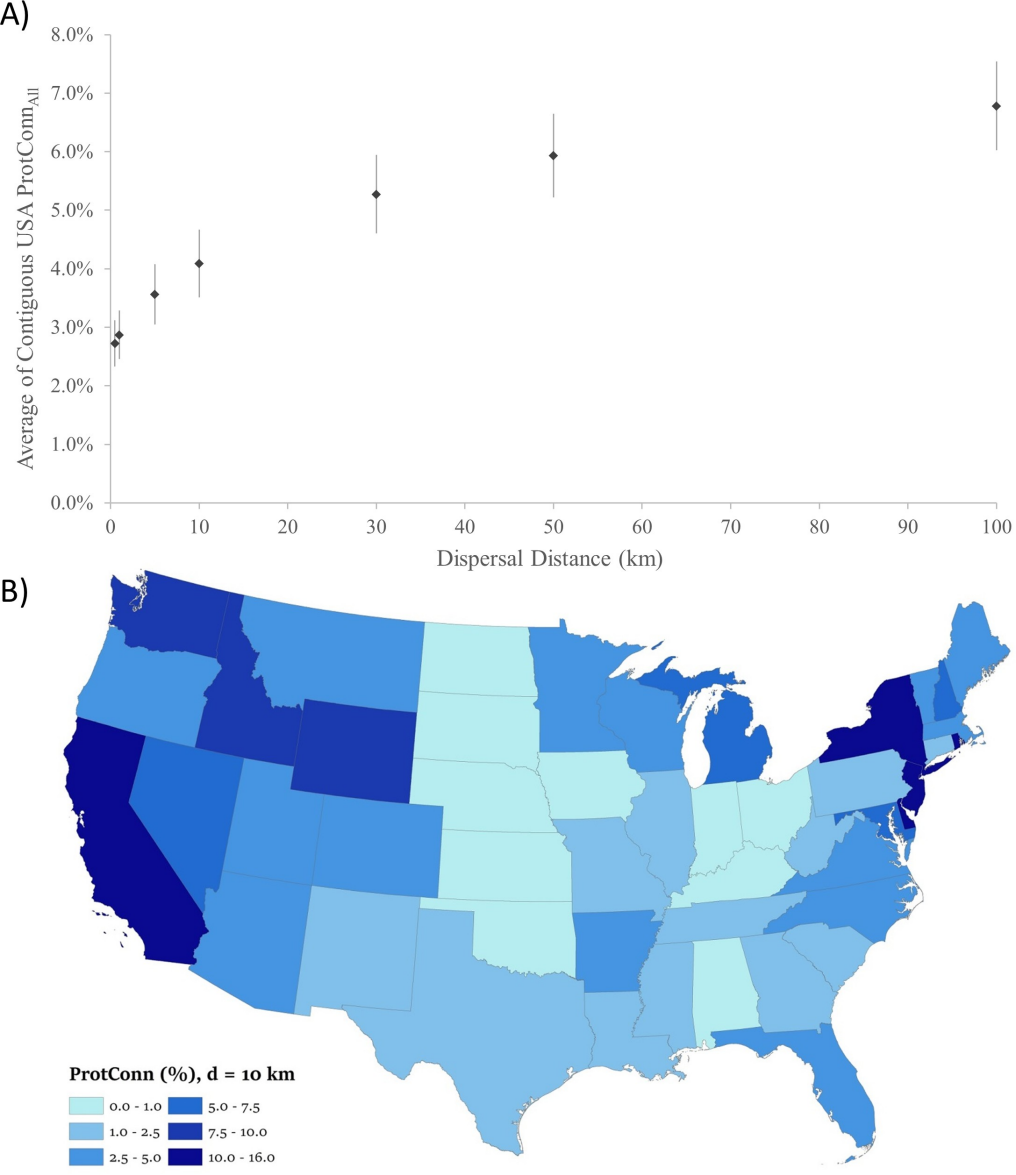
**A)** Average percent of protected and connected lands (ProtConn_All_), when considering all protected areas, for the contiguous United States, across seven dispersal distances (0.5 km, 1.0 km, 5.0 km, 10.0 km, 30.0 km, 50.0 km and 100.0 km). Standard error bars are *n* = 48 states. **B)** Percent of each state that is protected by a World Database of Protected Areas (WDPA) or National Conservation Easement Database (NCED) protected area patch and connected to another patch (ProtConn_All_) within a median dispersal (*d*) of 10 km.

Overall, the coverage and connectivity of protected areas was related to *d*, with average ProtConn_All_ ranging from 2.7% (*d =* 0.5 km) to 6.8% (*d* = 100.0 km) (Fig 1A). Protected area connectivity increased with dispersal distance and plateaued around 30.0 km. Average ProtConn_All_ for *d* = 10.0 km was 4.1% (range: 0.2% (Kansas); 15.8% (Delaware); Fig 1B), and the geographic pattern of ProtConn is comparable to percent state protected (S1 Fig). ProtConn_All_ increased with percent protected area coverage (S3 Fig).

Differences between percent of each state protected and ProtConn_All_ at *d* = 10.0 km (S4 Fig) highlight connectivity gaps in PANs. ProtConn_All_ >10.0% had the largest difference, indicating that even states with relatively high connectivity still have substantive gaps in protected area linkages. For example, California had a ProtConn_All_ of 13.6% and an overall 24.1% protected, resulting in 10.5% difference that may reflect lack of relation between ProtConn_All_ and number of protected patches (for all states: *R*^*2*^ = 0.02, S5 Fig), and median size of patches (for all states: *R*^*2*^ = 0.01, S6 Fig). Overall, states had a wide range in number and density of protected patches, ranging from New Mexico (*n =* 334 patches; 0.001/km^2^) to Minnesota (*n* = 11,929 patches; 0.055/km^2^), but Massachusetts had the highest density of patches (*n* = 5,653 patches; 0.266/km^2^). With a national average size of 42.3 ha for protected patches, Maryland had the smallest (6.8 ha, ProtConn_All_ = 5.9%) and New Mexico had the largest (256.6 ha, ProtConn_All_ = 1.5%) median area of patches. Percent of each state that is protected followed similar patterns (S8 and S9 Figs). Importantly, there was no clear relationship between number of privately protected patches and percent of a given state that is protected (S10 Fig).

**Fig 2:**
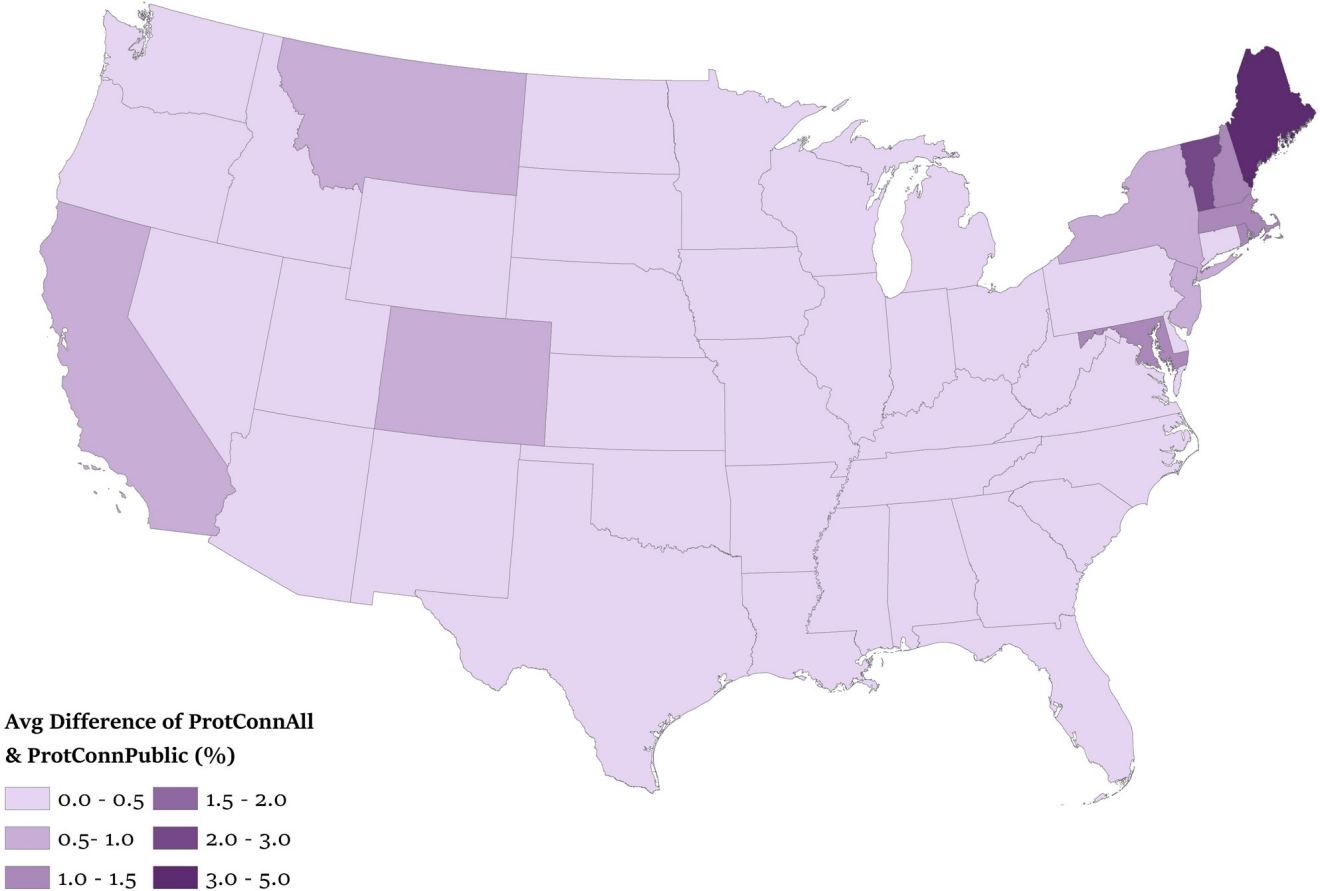
Difference between protected and connected patches (ProtConn_All_) and only publicly protected patches connected (ProtConn_Public_), averaged for seven dispersal distances in the contiguous United States.

We found little evidence that private protected areas contributed to PAN connectivity (Fig 2). When we calculated average difference between ProtConn_All_ and ProtConn_Public_ for all dispersal distances, Maine had the greatest contribution of privately-protected areas to connectivity (4.9%), followed by Vermont (2.8%), whereas Iowa and Nevada had the lowest (0.0%) (mean across all states: 0.4%, *n* = 48). This difference highlights the overall small contribution of private protected areas to connectivity of the PAN of each state; private protected areas increase average ProtConn_All_ by > 1% in only 6 of 48 states.

### Predictors of PAN coverage and connectivity

The four best-fit models predicting protected area coverage were statistically indistinguishable from each other (ΔAICc < 2.0). The top models for predicting protected area coverage include: (1) terrain ruggedness (estimate: 0.509 ± 0.131 SE, *p* < 0.001); (2) terrain ruggedness + per capita income (terrain ruggedness: estimate: 0.509 ± 0.130 SE, *p* < 0.001; per capita income: estimate: -0.187 ± 0.152 SE, *p* = 0.22); (3) percent farmland + population density (percent farmland: estimate: -0.368 ± 0.156 SE, *p* = 0.018; population density: estimate: -0.600 ± 0.123 SE, *p* < 0.001); and (4) terrain ruggedness + percent private (terrain ruggedness: estimate: 0.515 ± 0.132 SE, *p* < 0.001; percent private: estimate: -0.118 ± 0.117 SE, *p* = 0.31) (Table 1). Thus, private protected areas did not contribute notably to protected area coverage. For ProtConn_All_, the top model only included terrain ruggedness which was a positive driver for PAN connectivity (estimate: 0.576 ± 0.148 SE, *p* < 0.001); private protected areas did not influence ProtConn_All_ (Table 2).

**Table 1:** Modeling predictors of state protected areas.

	Parameter (*k*)	AICc	ΔAIC	*w*	McFadden *R*^*2*^
Terrain Ruggedness	3	1425.2	0	0.256	0.014
Terrain Ruggedness + Per Capita Income	4	1426.1	0.9	0.161	0.016
% Farmland + Population Density	4	1426.7	1.5	0.123	0.001
Terrain Ruggedness + % Private Protected	4	1426.7	1.5	0.121	0.013
Terrain Ruggedness + % Farmland	4	1427.6	2.4	0.078	0.016
Terrain Ruggedness * % Farmland	5	1427.7	2.5	0.075	0.016
% Farmland + Population Density + Per Capita Income	5	1427.8	2.6	0.070	0.016
Terrain Ruggedness + % Farmland + Per Capita Income	5	1428.6	3.4	0.047	0.008
Terrain Ruggedness + % Farmland + % Private Protected	5	1429.2	4.0	0.035	0.011
Population Density	3	1429.4	4.2	0.031	0.004

Top ten multi-variable models relating percent of each state that is protected relative to geographic, sociopolitical and economic factors for the contiguous United States. Twelve additional models, including a null model, were used in the modeling exercise but are excluded from the table due to low AIC weight (all *w* < 0.001).

**Table 2:** Modeling predictors of state ProtConn_All_.

	Parameter (*k*)	AICc	ΔAIC	*w*	McFadden *R*^*2*^
Terrain Ruggedness	3	1344.8	0	0.393	0.011
Terrain Ruggedness + % Private Protected	4	1347.0	2.3	0.126	0.011
Terrain Ruggedness * % Farmland	5	1347.1	2.3	0.125	0.013
Terrain Ruggedness + % Farmland	4	1347.1	2.3	0.125	0.011
Terrain Ruggedness + Per Capita Income	4	1347.1	2.4	0.119	0.011
Terrain Ruggedness + % Farmland + % Private Protected	5	1349.3	4.6	0.040	0.012
Terrain Ruggedness + % Farmland + Per Capita Income	5	1349.6	4.8	0.036	0.011
% Farmland + Population Density + Per Capita Income	5	1350.8	6.0	0.020	0.011
% Farmland + Population Density	4	1351.9	7.1	0.011	0.008
Population Density	3	1354.6	9.9	0.003	0.004

Top ten multi-variable models relating percent of each state that is protected and connected (ProtConn_All_) for *d* = 10 km relative to geographic, sociopolitical and economic factors for the contiguous United States. Twelve additional models, including a null model, were used in the modeling exercise but are excluded from the table due to low AIC weight (all *w* < 0.001).

## Discussion

By using states as individual units, this study revealed inconsistencies in PAN connectivity and the overall minor contribution of private conservation to PANs in the contiguous US. We found that number and size of protected areas did not relate directly to landscape connectivity of PANs or percent of land under protection in each state. In addition, protected areas occurred in areas with predictable landcover features, aligning with findings from previous studies [22,23].

Traditionally, protected area selection was largely opportunistic and an ad hoc process (for an illustrative example, see S13 Fig; McCune et al. [18]), but strategic placement of protected areas is necessary for well-connected PANs that facilitate multi-scale biological processes benefitting a wide variety of species [2,37]. Additionally, it is possible that larger tracts of land were prioritized over smaller, and potentially more strategic, parcels, as these have often been overlooked as critical for biodiversity conservation [39]. Overall, we found that the contiguous US is not on track to meet international targets for land protection [5] and that efforts should be increased to augment protected area coverage, with emphasis on strategic selection of private lands that will improve PAN connectivity.

It is important to recognize that the establishment and decommissioning of protected areas is a fluid process, with both occurring continually over time [44,45]. Recognizing that protected area coverage and connectivity may have changed since we collected data for this study (August 2017), it is likely that repeating our study in the foreseeable future will not alter our results qualitatively until there is a philosophical shift in the process of protected area selection.

Overall, mismatch between protected area coverage and conservation priorities is a prevalent problem in conservation planning [46], and PANs in the western US have overall better structural connectivity than their eastern counterparts. Although our PAN connectivity metric does not estimate overall landscape permeability (i.e., functional movement across it), both follow similar trends, with western states having higher connectivity because of a greater proportion of minimally-developed natural lands. Indeed, 51% and 2% of landcover in western and eastern states, respectively, is comprised of minimally-developed lands [9,17], thereby highlighting the restricted opportunities for establishing PANs in areas with intensive land use, often in areas that need them the most. In particular, the southeastern US is identified as a top conservation priority due to threats to high biological diversity [46], but to develop effective PANs in this region will require emphasis on integrating private lands that have strategic location, suitable coverage, and adequate protection.

The US has a long history of private land conservation, but internationally a substantive amount of land is privately protected through various conservation strategies [36]. Land tenure laws vary widely and political bodies have inconsistent legal frameworks, preventing adoption of an easy one-size-fits-all approach towards private land conservation. Although gaps in protected area data limit a consensus on how well these areas contribute to protected area connectivity, private protected areas are recognized globally as a biodiversity conservation priority [5,36,47]. Indeed, the influence of private protected areas on PAN connectivity in eastern states, like Maine and Vermont, is reliant on an effective system of conservation easements or land donations. While >90% of Maine’s land area is privately owned, roughly 9.2% of the state is held in conservation easements. This strategy is an effective planning tool that can contribute to long-term land protection, and is gaining support internationally [48]. However, for many states and other countries, the lack of legal framework to incentivize landowners to place restrictions on their private property doubtless precludes widespread use of this mechanism in conservation planning. Likewise, current strategic planning decisions rarely evaluate private lands in the context of their contribution to PAN connectivity, despite that such inclusion is crucial in ensuring ecosystem functionality [18,49].

Protecting strategically important private land is not the only mechanism for effective conservation planning, but ultimately it should contribute substantially to mitigating biodiversity loss in highly-fragmented landscapes and thereby help reach Aichi Target 11 objectives [50]. Recent evidence of trends to legally downgrade or downsize protected areas in the United States further highlights the need for diversified conservation strategies [45]. In the absence of robust national policy to help guide these goals, state, regional, or municipal policies must consider strategic conservation planning decisions that will help fill gaps in PAN coverage and connectivity. It is only through such important and concerted efforts that we may potentially reach our global targets for biodiversity conservation, and thereby help mitigate ongoing and anticipated losses of natural areas, biodiversity, and ecosystem services.

## Supporting information

S1 Table Protected land feature metrics by state for the contiguous United States. ProtConn_All_ represents the percentage of land that is protected by all designated protected areas within the given dispersal distance and ProtConn_Public_ is the metric with only publicly designated protected areas. ProtConn_All_ is given across 7 dispersal distances. Data were obtained from the World Database of Protected Areas (http://www.protectedplanet.net/, accessed August 2017) (WDPA) and the National Conservation Easement Database (https://www.conservationeasement.us/, accessed August 2017) (NCED).
(DOCX)

S2 Table Difference between percent protected of each state in the contiguous United States versus its ProtConn_All_, defined as the percent of each state protected and connected within set dispersal distance. ProtConn_All_ values are standardized by the area of each state, across seven dispersal distances. Data were obtained from WDPA (2017) and NCED (2017).
(DOCX)

S3 Table Geographic, economic and sociopolitical factors used in multivariate models to determine predictors of the percent of each state in the contiguous United States that is protected and the percent of protected and connected land (ProtConn_All_, *d* = 10 km). (DOCX)

S4 Table Top ten multivariate models relating the percent of each state in the contiguous United States that is protected and connected (ProtConn_All_) for *d* = 100 km, relative to geographic, sociopolitical and economic factors. Twelve additional models, including a null model, were used in the modeling exercise but are exclude from the table due to low AIC weight (all *w* < 0.001).
(DOCX)

S1 Fig Percent of each state that is covered by a World Database of Protected Areas (WDPA) or National Conservation Easement Database (NCED) protected area. The average for the contiguous United States, by state, is 8.4% (range: 0.7% (Kansas), 24.1% (California)).
(TIF)

S2 Fig Percent of each state in the contiguous United States that is covered by a protected area that is managed privately. The average, by state, is 1.1% (range: 0.0% (Nevada) to 9.2% (Maine)).
(TIF)

S3 Fig Relationship between the percent of each state in the contiguous United States that is protected by public and private protected areas and the percent of each state that is protected and connected (ProtConn_All_) at a median dispersal of 10 km. (JPG)

S4 Fig The area-standardized difference between land that is protected and connected (ProtConn_All_) for a median dispersal distance (*d =* 10 km) and the total percent of each state that is protected, for the contiguous United States. (TIF)

S5 Fig Relationship between the number of protected area (PA) patches (after merging and dissolving the World Database of Protected Areas and National Conservation Easement datasets) and the percentage of protected and connected lands (ProtConn_All_) at a median dispersal distance of 10 km, for the contiguous United States. (JPG)

S6 Fig Relationship between median size of all protected area (PA) patches and the percentage of protected and connected lands (ProtConn_All_) at a median dispersal distance of 10 km, for the contiguous United States. (JPG)

S7 Fig Relationship between the number of protected area patches (after merging and dissolving the World Database of Protected Areas and National Conservation Easement Database datasets) and the percent of each state that is protected, for the contiguous United States. (JPG)

S8 Fig Relationship between the median size of all protected area patches and the total percentage of each state that is protected, for the contiguous United States. (JPG)

S9 Fig Relationship between the number of privately protected area (PA) patches and the percentage of protected and connected lands at a median dispersal distance of 10 km, for the contiguous United States. (JPG)

S10 Fig Relationship between the number of privately protected area (PA) patches and the total percent of each state that is protected, for the contiguous United States. (JPG)

S11 Fig Median area in hectares of all protected area patches for the contiguous United States. (TIF)

S12 Fig Correlation matrix of variables used in multivariate models to predict percent of state protected and ProtConn_All_, for the contiguous United States. Predictors that were removed after testing for collinearity (*r* > 0.70) are: average land value, average farm value, the number of land trusts, and the age of the oldest land trust.
(TIF)

S13 Fig Protected area (circled in red) in Texas demonstrates how a protected area could have been more strategically placed (where the blue star is) in a landscape context. For illustrative purposes only, as the ulterior motive for designating this area is unknown, the alternative placement would have served as a stepping stone between patches of protected areas.
(TIF)
