# Supplementary material for: Assessing connectivity and the contribution of private lands to protected area networks in the United States
Source: PLoS One. 2020 Mar 5;15(3):e0228946. doi: 10.1371/journal.pone.0228946 (PMC7058307; doi:10.1371/journal.pone.0228946)
Supplement: S1 Table — ProtConnAll represents the percentage of land that is protected by all designated protected areas within the given dispersal distance and ProtConnPublic is the metric with only publicly designated protected areas. ProtConnAll is given across 7 dispersal distances. Data were obtained from the World Database of Protected Areas (http://www.protectedplanet.net/, accessed August 2017) (WDPA) and the National Conservation Easement Database (https://www.conservationeasement.us/, accessed August 2017) (NCED). (DOCX) [file pone.0228946.s001.docx]

**Table S1.** Protected land feature metrics by state for the contiguous United States. ProtConn_All_ represents the percentage of land that is protected by all designated protected areas within the given dispersal distance and ProtConn_Public_ is the metric with only publicly designated protected areas. ProtConn_All_ is given across 7 dispersal distances. Data were obtained from the World Database of Protected Areas (http://www.protectedplanet.net/, accessed August 2017) (WDPA) and the National Conservation Easement Database (https://www.conservationeasement.us/, accessed August 2017) (NCED).

| **State** | **% Protected** | **% Private Protected** | **Average difference of ProtConn_All_ &ProtConn_Public_** | **ProtConn_All_ 0.50 km** | **ProtConn_All_ 1 km** | **ProtConn_All_ 5 km** | **ProtConn_All_**  **10 km** | **ProtConn_All_ 30 km** | **ProtConn_All_**  **50 km** | **ProtConn_All_ 100 km** |
| --- | --- | --- | --- | --- | --- | --- | --- | --- | --- | --- |
| AL | 2.25% | 0.41% | 0.10% | 0.39% | 0.42% | 0.48% | 0.56% | 0.81% | 1.02% | 1.38% |
| AZ | 10.15% | 0.09% | 0.02% | 2.88% | 2.90% | 2.98% | 3.15% | 4.26% | 5.30% | 6.90% |
| AR | 9.08% | 0.22% | 0.04% | 4.51% | 4.60% | 4.81% | 4.95% | 5.76% | 6.48% | 7.45% |
| CA | 24.08% | 2.00% | 0.75% | 7.64% | 8.35% | 11.54% | 13.58% | 16.78% | 18.38% | 20.44% |
| CO | 10.76% | 1.85% | 0.58% | 1.81% | 1.87% | 2.46% | 3.28% | 5.79% | 7.09% | 8.55% |
| CT | 3.62% | 0.96% | 0.48% | 0.50% | 0.57% | 1.00% | 1.44% | 2.39% | 2.78% | 3.15% |
| DE | 18.74% | 0.33% | 0.10% | 8.82% | 10.07% | 14.14% | 15.83% | 17.57% | 18.00% | 18.36% |
| FL | 11.61% | 1.58% | 0.13% | 3.71% | 3.73% | 3.87% | 4.15% | 5.51% | 6.61% | 8.23% |
| GA | 5.66% | 0.56% | 0.28% | 1.32% | 1.35% | 1.49% | 1.69% | 2.41% | 2.94% | 3.77% |
| ID | 14.13% | 0.08% | 0.01% | 7.73% | 7.74% | 7.95% | 8.23% | 9.52% | 10.53% | 11.87% |
| IL | 2.86% | 0.62% | 0.20% | 0.46% | 0.52% | 0.82% | 1.05% | 1.49% | 1.75% | 2.12% |
| IN | 2.92% | 0.22% | 0.05% | 0.50% | 0.57% | 0.79% | 0.99% | 1.52% | 1.80% | 2.17% |
| IA | 1.39% | 0.03% | 0% | 0.12% | 0.13% | 0.18% | 0.23% | 0.42% | 0.58% | 0.83% |
| KS | 0.73% | 0.11% | 0.03% | 0.12% | 0.12% | 0.13% | 0.15% | 0.23% | 0.31% | 0.43% |
| KY | 2.07% | 0.24% | 0.03% | 0.49% | 0.49% | 0.52% | 0.57% | 0.83% | 1.03% | 1.34% |
| LA | 6.10% | 0.32% | 0.12% | 1.13% | 1.17% | 1.53% | 2.00% | 3.18% | 3.84% | 4.66% |
| ME | 13.83% | 9.19% | 4.91% | 5.30% | 5.77% | 7.17% | 8.06% | 10.00% | 11.01% | 12.15% |
| MD | 13.89% | 2.20% | 1.13% | 1.74% | 2.12% | 4.12% | 5.90% | 9.44% | 10.83% | 12.15% |
| MA | 9.67% | 3.37% | 1.29% | 1.15% | 1.29% | 2.38% | 3.40% | 5.59% | 6.65% | 7.85% |
| MI | 11.98% | 0.22% | 0.12% | 5.42% | 5.48% | 5.66% | 5.81% | 6.83% | 7.77% | 9.18% |
| MN | 7.74% | 0.12% | 0.03% | 2.00% | 2.09% | 2.43% | 2.66% | 3.43% | 4.15% | 5.31% |
| MS | 4.33% | 0.60% | 0.18% | 0.70% | 0.75% | 1.02% | 1.21% | 1.79% | 2.22% | 2.90% |
| MO | 3.30% | 0.09% | 0.03% | 0.52% | 0.54% | 0.67% | 0.80% | 1.26% | 1.61% | 2.15% |
| MT | 10.45% | 1.71% | 0.78% | 3.54% | 3.60% | 4.10% | 4.45% | 5.69% | 6.69% | 8.05% |
| NE | 1.11% | 0.31% | 0.11% | 0.25% | 0.25% | 0.27% | 0.30% | 0.40% | 0.50% | 0.67% |
| NV | 15.27% | 0.02% | 0% | 4.44% | 4.70% | 5.91% | 6.48% | 7.83% | 8.89% | 10.73% |
| NH | 14.15% | 4.75% | 1.5% | 3.50% | 3.66% | 4.86% | 6.15% | 9.28% | 10.71% | 12.19% |
| NJ | 22.28% | 0.83% | 0.53% | 8.83% | 9.43% | 12.63% | 15.03% | 18.71% | 19.93% | 21.01% |
| NM | 5.53% | 0.36% | 0.09% | 1.22% | 1.26% | 1.38% | 1.49% | 2.11% | 2.70% | 3.60% |
| NY | 13.12% | 1.37% | 0.87% | 10.19% | 10.32% | 10.61% | 10.75% | 11.20% | 11.55% | 12.07% |
| NC | 5.53% | 0.98% | 0.31% | 2.15% | 2.22% | 2.46% | 2.68% | 3.14% | 3.38% | 3.83% |
| ND | 2.39% | 0.04% | 0.05% | 0.26% | 0.26% | 0.31% | 0.41% | 0.80% | 1.10% | 1.53% |
| OH | 1.15% | 0.31% | 0.08% | 0.11% | 0.12% | 0.18% | 0.24% | 0.42% | 0.55% | 0.75% |
| OK | 2.41% | 0.17% | 0.04% | 0.34% | 0.36% | 0.43% | 0.53% | 0.86% | 1.11% | 1.49% |
| OR | 11.61% | 0.18% | 0.04% | 2.82% | 2.99% | 4.06% | 4.74% | 6.23% | 7.31% | 8.83% |
| PA | 4.30% | 0.84% | 0.30% | 0.39% | 0.43% | 0.70% | 1.01% | 1.88% | 2.41% | 3.11% |
| RI | 14.80% | 3.34% | 1.39% | 6.15% | 6.86% | 9.84% | 11.40% | 13.31% | 13.84% | 14.29% |
| SC | 4.94% | 1.21% | 0.44% | 1.13% | 1.21% | 1.60% | 1.91% | 2.74% | 3.21% | 3.81% |
| SD | 2.20% | 0.16% | 0.08% | 0.59% | 0.59% | 0.65% | 0.71% | 0.92% | 1.08% | 1.35% |
| TN | 4.09% | 0.42% | 0.11% | 1.18% | 1.20% | 1.28% | 1.40% | 1.86% | 2.19% | 2.71% |
| TX | 3.15% | 0.27% | 0.02% | 1.77% | 1.78% | 1.79% | 1.80% | 1.85% | 1.91% | 2.05% |
| UT | 11.79% | 0.11% | 0.02% | 2.32% | 2.39% | 3.26% | 4.22% | 6.44% | 7.64% | 9.16% |
| VT | 10.32% | 6.25% | 2.78% | 1.52% | 1.71% | 2.90% | 4.11% | 6.75% | 7.85% | 8.93% |
| VA | 8.98% | 0.91% | 0.29% | 1.71% | 1.88% | 2.90% | 3.70% | 5.51% | 6.42% | 7.45% |
| WA | 15.32% | 0.27% | 0.09% | 6.32% | 6.54% | 8.15% | 9.16% | 11.27% | 12.32% | 13.51% |
| WV | 4.40% | 0.08% | 0.02% | 0.81% | 0.83% | 1.02% | 1.25% | 2.10% | 2.63% | 3.30% |
| WI | 8.82% | 0.36% | 0.20% | 2.87% | 3.00% | 3.59% | 4.29% | 5.89% | 6.68% | 7.55% |
| WY | 11.74% | 0.99% | 0.29% | 7.46% | 7.49% | 8.08% | 8.48% | 9.18% | 9.63% | 10.30% |
