# Supplementary material for: Assessing connectivity and the contribution of private lands to protected area networks in the United States
Source: PLoS One. 2020 Mar 5;15(3):e0228946. doi: 10.1371/journal.pone.0228946 (PMC7058307; doi:10.1371/journal.pone.0228946)
Supplement: S2 Table — ProtConnAll values are standardized by the area of each state, across seven dispersal distances. Data were obtained from WDPA (2017) and NCED (2017). (DOCX) [file pone.0228946.s002.docx]

**Table S2.** Difference between percent protected of each state in the contiguous United States versus its ProtConn_All_, defined as the percent of each state protected and connected within set dispersal distance. ProtConn_All_ values are standardized by the area of each state, across seven dispersal distances. Data were obtained from WDPA (2017) and NCED (2017).

| **State** | **Area standardized difference of %Protected & ProtConn_All, 0.5km_** | **Area standardized difference of %Protected & ProtConn_All, 1km_** | **Area standardized difference of %Protected & ProtConn_All, 5km_** | **Area standardized difference of %Protected & ProtConn_All, 10km_** | **Area standardized difference of %Protected & ProtConn_All, 30km_** | **Area standardized difference of %Protected & ProtConn_All, 50km_** | **Area standardized difference of %Protected & ProtConn_All, 100km_** |
| --- | --- | --- | --- | --- | --- | --- | --- |
| AL | 1.39E-09 | 1.37E-09 | 1.32E-09 | 1.26E-09 | 1.08E-09 | 9.21E-10 | 6.54E-10 |
| AZ | 2.46E-09 | 2.46E-09 | 2.43E-09 | 2.37E-09 | 2.00E-09 | 1.64E-09 | 1.10E-09 |
| AR | 3.32E-09 | 3.25E-09 | 3.10E-09 | 2.99E-09 | 2.41E-09 | 1.89E-09 | 1.19E-09 |
| CA | 4.01E-09 | 3.84E-09 | 3.06E-09 | 2.56E-09 | 1.78E-09 | 1.39E-09 | 8.90E-10 |
| CO | 3.32E-09 | 3.30E-09 | 3.08E-09 | 2.77E-09 | 1.84E-09 | 1.36E-09 | 8.19E-10 |
| CT | 2.41E-08 | 2.36E-08 | 2.02E-08 | 1.68E-08 | 9.52E-09 | 6.53E-09 | 3.65E-09 |
| DE | 1.90E-07 | 1.66E-07 | 8.84E-08 | 5.58E-08 | 2.26E-08 | 1.42E-08 | 7.35E-09 |
| FL | 5.23E-09 | 5.22E-09 | 5.12E-09 | 4.94E-09 | 4.04E-09 | 3.31E-09 | 2.24E-09 |
| GA | 2.84E-09 | 2.83E-09 | 2.73E-09 | 2.60E-09 | 2.13E-09 | 1.78E-09 | 1.24E-09 |
| ID | 2.96E-09 | 2.95E-09 | 2.86E-09 | 2.72E-09 | 2.13E-09 | 1.66E-09 | 1.04E-09 |
| IL | 1.64E-09 | 1.60E-09 | 1.40E-09 | 1.24E-09 | 9.39E-10 | 7.59E-10 | 5.04E-10 |
| IN | 2.58E-09 | 2.51E-09 | 2.27E-09 | 2.05E-09 | 1.49E-09 | 1.20E-09 | 8.05E-10 |
| IA | 8.76E-10 | 8.69E-10 | 8.33E-10 | 7.99E-10 | 6.65E-10 | 5.58E-10 | 3.87E-10 |
| KS | 2.87E-10 | 2.85E-10 | 2.79E-10 | 2.71E-10 | 2.32E-10 | 1.97E-10 | 1.39E-10 |
| KY | 1.51E-09 | 1.50E-09 | 1.48E-09 | 1.42E-09 | 1.18E-09 | 9.90E-10 | 6.92E-10 |
| LA | 4.07E-09 | 4.04E-09 | 3.74E-09 | 3.35E-09 | 2.39E-09 | 1.85E-09 | 1.18E-09 |
| ME | 1.00E-08 | 9.50E-09 | 7.84E-09 | 6.79E-09 | 4.51E-09 | 3.32E-09 | 1.98E-09 |
| MD | 4.56E-08 | 4.42E-08 | 3.67E-08 | 2.99E-08 | 1.67E-08 | 1.15E-08 | 6.51E-09 |
| MA | 4.01E-08 | 3.94E-08 | 3.43E-08 | 2.94E-08 | 1.92E-08 | 1.42E-08 | 8.53E-09 |
| MI | 4.34E-09 | 4.30E-09 | 4.18E-09 | 4.08E-09 | 3.41E-09 | 2.78E-09 | 1.85E-09 |
| MN | 2.63E-09 | 2.59E-09 | 2.43E-09 | 2.32622E-09 | 1.98E-09 | 1.64E-09 | 1.11E-09 |
| MS | 2.94E-09 | 2.90E-09 | 2.69E-09 | 2.52828E-09 | 2.06E-09 | 1.71E-09 | 1.16E-09 |
| MO | 1.54E-09 | 1.53E-09 | 1.46E-09 | 1.38444E-09 | 1.13E-09 | 9.36E-10 | 6.35E-10 |
| MT | 1.82E-09 | 1.80E-09 | 1.67E-09 | 1.5767E-09 | 1.25E-09 | 9.90E-10 | 6.33E-10 |
| NE | 4.32E-10 | 4.28E-10 | 4.19E-10 | 4.05888E-10 | 3.54E-10 | 3.07E-10 | 2.23E-10 |
| NV | 3.78E-09 | 3.69E-09 | 3.27E-09 | 3.06807E-09 | 2.60E-09 | 2.23E-09 | 1.58E-09 |
| NH | 4.43E-08 | 4.36E-08 | 3.86E-08 | 3.32768E-08 | 2.02E-08 | 1.43E-08 | 8.17E-09 |
| NJ | 6.68E-08 | 6.38E-08 | 4.79E-08 | 3.6006E-08 | 1.77E-08 | 1.17E-08 | 6.32E-09 |
| NM | 1.37E-09 | 1.35E-09 | 1.32E-09 | 1.28169E-09 | 1.08E-09 | 8.99E-10 | 6.11E-10 |
| NY | 2.31E-09 | 2.20E-09 | 1.98E-09 | 1.86737E-09 | 1.52E-09 | 1.24E-09 | 8.27E-10 |
| NC | 2.62E-09 | 2.57E-09 | 2.39E-09 | 2.21498E-09 | 1.86E-09 | 1.67E-09 | 1.32E-09 |
| ND | 1.17E-09 | 1.16E-09 | 1.14E-09 | 1.08263E-09 | 8.71E-10 | 7.07E-10 | 4.71E-10 |
| OH | 9.79E-10 | 9.69E-10 | 9.14E-10 | 8.56165E-10 | 6.86E-10 | 5.64E-10 | 3.83E-10 |
| OK | 1.14E-09 | 1.14E-09 | 1.09E-09 | 1.04001E-09 | 8.57E-10 | 7.22E-10 | 5.11E-10 |
| OR | 3.50E-09 | 3.43E-09 | 3.00E-09 | 2.73204E-09 | 2.14E-09 | 1.71E-09 | 1.10E-09 |
| PA | 3.33E-09 | 3.30E-09 | 3.07E-09 | 2.80426E-09 | 2.06E-09 | 1.61E-09 | 1.02E-09 |
| RI | 3.04E-07 | 2.79E-07 | 1.74E-07 | 1.19605E-07 | 5.27E-08 | 3.39E-08 | 1.82E-08 |
| SC | 4.73E-09 | 4.63E-09 | 4.15E-09 | 3.7654E-09 | 2.73E-09 | 2.15E-09 | 1.41E-09 |
| SD | 8.06E-10 | 8.02E-10 | 7.75E-10 | 7.43905E-10 | 6.36E-10 | 5.61E-10 | 4.25E-10 |
| TN | 2.66E-09 | 2.65E-09 | 2.57E-09 | 2.46039E-09 | 2.04E-09 | 1.73E-09 | 1.26E-09 |
| TX | 2.00E-10 | 1.99E-10 | 1.97E-10 | 1.95833E-10 | 1.89E-10 | 1.80E-10 | 1.60E-10 |
| UT | 4.31E-09 | 4.28E-09 | 3.88E-09 | 3.44331E-09 | 2.44E-09 | 1.89E-09 | 1.19E-09 |
| VT | 3.53E-08 | 3.46E-08 | 2.98E-08 | 2.49511E-08 | 1.43E-08 | 9.93E-09 | 5.59E-09 |
| VA | 6.93E-09 | 6.77E-09 | 5.80E-09 | 5.03162E-09 | 3.31E-09 | 2.43E-09 | 1.45E-09 |
| WA | 5.13E-09 | 5.01E-09 | 4.09E-09 | 3.5123E-09 | 2.31E-09 | 1.71E-09 | 1.04E-09 |
| WV | 5.72E-09 | 5.69E-09 | 5.38E-09 | 5.01803E-09 | 3.66E-09 | 2.81E-09 | 1.76E-09 |
| WI | 4.10E-09 | 4.01E-09 | 3.60E-09 | 3.11716E-09 | 2.02E-09 | 1.48E-09 | 8.76E-10 |
| WY | 1.69E-09 | 1.68E-09 | 1.44E-09 | 1.28632E-09 | 1.01E-09 | 8.33E-10 | 5.68E-10 |
