# Supplementary material for: Assessing connectivity and the contribution of private lands to protected area networks in the United States
Source: PLoS One. 2020 Mar 5;15(3):e0228946. doi: 10.1371/journal.pone.0228946 (PMC7058307; doi:10.1371/journal.pone.0228946)
Supplement: S3 Table — (DOCX) [file pone.0228946.s003.docx]

**Table S3.** Geographic, economic and sociopolitical factors used in multivariate models to determine predictors of the percent of each state in the contiguous United States that is protected and the percent of protected and connected land (ProtConn_All_, *d* = 10 km).

| **State** | **Average Terrain Ruggedness Index (m)^1^** | **Farmland (%)^2^** | **Per Capita Income ($)^3^** | **Population Density (/sqkm)^4^** | **Median Land Trust Age (yrs)^5^** | **Politics^6^** | **Private Protected Areas (%)** |
| --- | --- | --- | --- | --- | --- | --- | --- |
| AL | 45.5 | 27.5 | 38030 | 37 | 26 | Red | 0.48 |
| AZ | 123.3 | 36.1 | 39156 | 23 | 21 | Red | 0.09 |
| AR | 50.3 | 41.5 | 38252 | 22 | 10 | Red | 0.22 |
| CA | 209.4 | 25.6 | 53741 | 97 | 24 | Blue | 2.00 |
| CO | 159.7 | 48.1 | 50899 | 20 | 23 | Blue | 1.85 |
| CT | 75.1 | 14.1 | 68704 | 286 | 42 | Blue | 0.96 |
| DE | 5.6 | 40.8 | 47633 | 187 | 38 | Blue | 0.33 |
| FL | 7.2 | 27.8 | 44429 | 145 | 26 | Red | 1.58 |
| GA | 36.2 | 26.1 | 40306 | 68 | 8 | Red | 0.56 |
| ID | 240.5 | 22.2 | 38392 | 7 | 20 | Red | 0.08 |
| IL | 17.5 | 75.8 | 50295 | 89 | 27 | Blue | 0.62 |
| IN | 21.0 | 64.2 | 41940 | 87 | 21 | Red | 0.22 |
| IA | 28.1 | 85.7 | 45902 | 21 | 37 | Blue | 0.03 |
| KS | 21.6 | 88.2 | 47161 | 14 | 17 | Red | 0.11 |
| KY | 80.7 | 51.6 | 38588 | 43 | 20 | Red | 0.24 |
| LA | 9.4 | 28.6 | 42947 | 41 | 13 | Red | 0.32 |
| ME | 80.1 | 7.4 | 42799 | 16 | 29 | Blue | 9.19 |
| MD | 42.5 | 32.5 | 55972 | 238 | 24 | Blue | 2.20 |
| MA | 66.5 | 10.5 | 62603 | 336 | 31 | Blue | 3.37 |
| MI | 23.3 | 27.5 | 42812 | 67 | 24 | Blue | 0.22 |
| MN | 18.0 | 51.1 | 50871 | 26 | 25 | Blue | 0.12 |
| MS | 23.0 | 36.4 | 34771 | 24 | 15 | Red | 0.60 |
| MO | 37.9 | 64.2 | 42300 | 34 | 23 | Red | 0.09 |
| MT | 147.7 | 64.2 | 41809 | 2 | 23 | Red | 1.71 |
| NE | 28.0 | 92.2 | 48544 | 9 | 33 | Red | 0.31 |
| NV | 152.8 | 8.4 | 41889 | 10 | 24 | Blue | 0.02 |
| NH | 149.7 | 8.3 | 55905 | 57 | 27 | Blue | 4.75 |
| NJ | 33.6 | 15.2 | 59949 | 470 | 27 | Blue | 0.83 |
| NM | 88.1 | 55.6 | 37938 | 6 | 19 | Blue | 0.36 |
| NY | 95.5 | 23.8 | 58670 | 162 | 25 | Red | 1.37 |
| NC | 62.5 | 27 | 40759 | 79 | 22 | Red | 0.98 |
| ND | 21.9 | 88.9 | 55950 | 4 | 0 | Red | 0.04 |
| OH | 32.3 | 53.4 | 43566 | 109 | 19 | Red | 0.31 |
| OK | 32.0 | 78.3 | 45573 | 22 | 14 | Red | 0.17 |
| OR | 188.1 | 26.5 | 43783 | 16 | 21 | Blue | 0.18 |
| PA | 115.2 | 26.9 | 49746 | 110 | 25 | Blue | 0.84 |
| RI | 37.8 | 10.5 | 50018 | 394 | 28 | Blue | 3.34 |
| SC | 24.3 | 25.8 | 38302 | 62 | 24 | Red | 1.21 |
| SD | 31.0 | 89.2 | 47881 | 4 | 22 | Red | 0.16 |
| TN | 83.1 | 41.2 | 42094 | 61 | 24 | Red | 0.42 |
| TX | 31.7 | 77.8 | 46947 | 40 | 20 | Red | 0.27 |
| UT | 176.2 | 20.9 | 39308 | 14 | 23 | Red | 0.11 |
| VT | 162.3 | 21.2 | 48587 | 26 | 26 | Blue | 6.25 |
| VA | 95.0 | 32.8 | 52052 | 26 | 22 | Blue | 0.91 |
| WA | 256.9 | 34.7 | 51898 | 41 | 26 | Blue | 0.27 |
| WV | 172.0 | 23.4 | 36758 | 29 | 22 | Red | 0.08 |
| WI | 33.1 | 42 | 45914 | 41 | 22 | Blue | 0.36 |
| WY | 118.7 | 48.9 | 56081 | 2 | 16 | Red | 0.99 |

^1^ Riley, S. J., DeGloria, S. D., & Elliot, R. (1999). A Terrain Ruggedness Index that Quantifies Topographic Heterogeneity. *Intermountain Journal of Sciences*, *5*(1–4), 23–27. http://doi.org/citeulike-article-id:8858430

^2^United States Department of Agriculture, National Agricultural Statistics Service. (2013). *Table 1. State Summary Highlights: 2012. 2012 Census of Agriculture.* Retrieved from

https://www.agcensus.usda.gov/Publications/2012/Full_Report/Volume_1,_Chapter_2_US_State_Level/st99_2_001_001.pdf

^3^Bureau of Economic Analysis. (2017, September 26). *Regional Data: SA1 Personal Income Summary: Personal Income, Population, Per Capita Personal Income.* Retrieved from www.bea.gov/itable

^4^United States Census Bureau. (2011) Resident Population Data (Text Version). United States Census 2010. Retrieved from https://www.census.gov/2010census/data/apportionment-dens-text.php

^5^Land Trust Alliance. (2016) *National Land Trust Census*. Retrieved from. https://www.landtrustalliance.org/census-map

^6^270towin.com. (2016*). Historical presidential election information by state*. Retrieved from https://www.270towin.com/states
