# Supplementary material for: Assessing connectivity and the contribution of private lands to protected area networks in the United States
Source: PLoS One. 2020 Mar 5;15(3):e0228946. doi: 10.1371/journal.pone.0228946 (PMC7058307; doi:10.1371/journal.pone.0228946)
Supplement: S4 Table — Twelve additional models, including a null model, were used in the modeling exercise but are exclude from the table due to low AIC weight (all w < 0.001). (DOCX) [file pone.0228946.s004.docx]

**Table S4.** Top ten multivariate models relating the percent of each state in the contiguous United States that is protected and connected (ProtConn_All_) for *d* = 100 km, relative to geographic, sociopolitical and economic factors. Twelve additional models, including a null model, were used in the modeling exercise but are exclude from the table due to low AIC weight (all *w* < 0.001).

|  | **Parameter (*k*)** | **AICc** | Δ**AIC** | | ***w*** | **McFadden *R^2^*** |
| --- | --- | --- | --- | --- | --- | --- |
| Terrain Ruggedness | 3 | 1397.0 | | 0 | 0.334 | 0.012 |
| Terrain Ruggedness * Farmland | 5 | 1398.4 | | 1.4 | 0.166 | 0.015 |
| Terrain Ruggedness + Per Capita Income | 4 | 1398.8 | | 1.9 | 0.132 | 0.013 |
| Terrain Ruggedness + % Private Protected | 4 | 1399.0 | | 2.0 | 0.121 | 0.012 |
| Terrain Ruggedness + % Farmland | 4 | 1399.3 | | 2.3 | 0.1054 | 0.012 |
| Terrain Ruggedness + % Farmland + Per Capita Income | 5 | 1401.3 | | 4.3 | 0.039 | 0.013 |
| Terrain Ruggedness + % Farmland + % Private | 5 | 1401.3 | | 4.3 | 0.039 | 0.013 |
| % Farmland + Population Density + Per Capita Income | 5 | 1401.9 | | 4.9 | 0.0289 | 0.012 |
| % Farmland + Population Density | 4 | 1401.9 | | 4.9 | 0.029 | 0.010 |
| Population Density | 3 | 1405.7 | | 8.7 | 0.004 | 0.006 |
