# Supplementary figures and images for: Assessing connectivity and the contribution of private lands to protected area networks in the United States
Source: PLoS One. 2020 Mar 5;15(3):e0228946. doi: 10.1371/journal.pone.0228946 (PMC7058307; doi:10.1371/journal.pone.0228946)

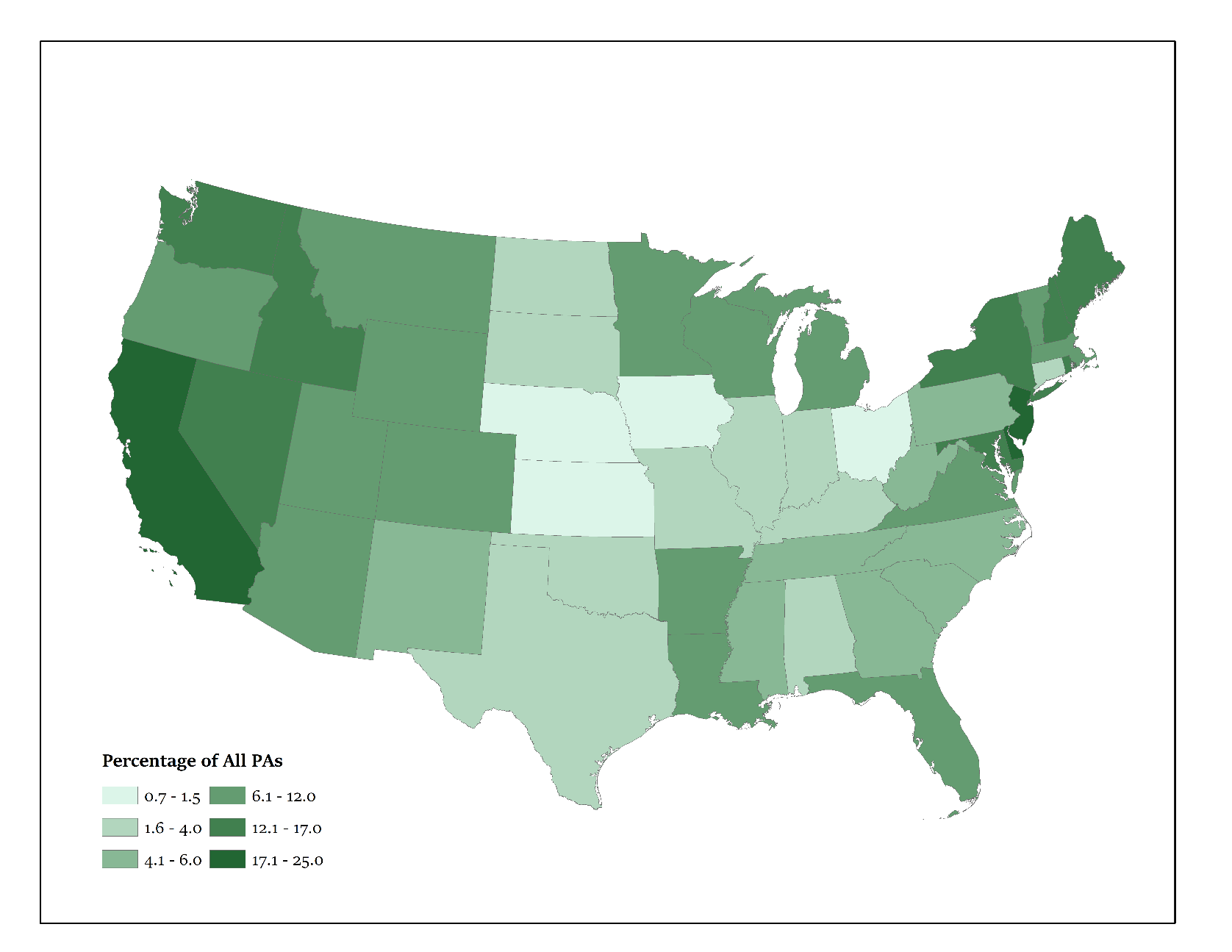

Supplement: S1 Fig — The average for the contiguous United States, by state, is 8.4% (range: 0.7% (Kansas), 24.1% (California)). (TIF) [file pone.0228946.s005.tif]

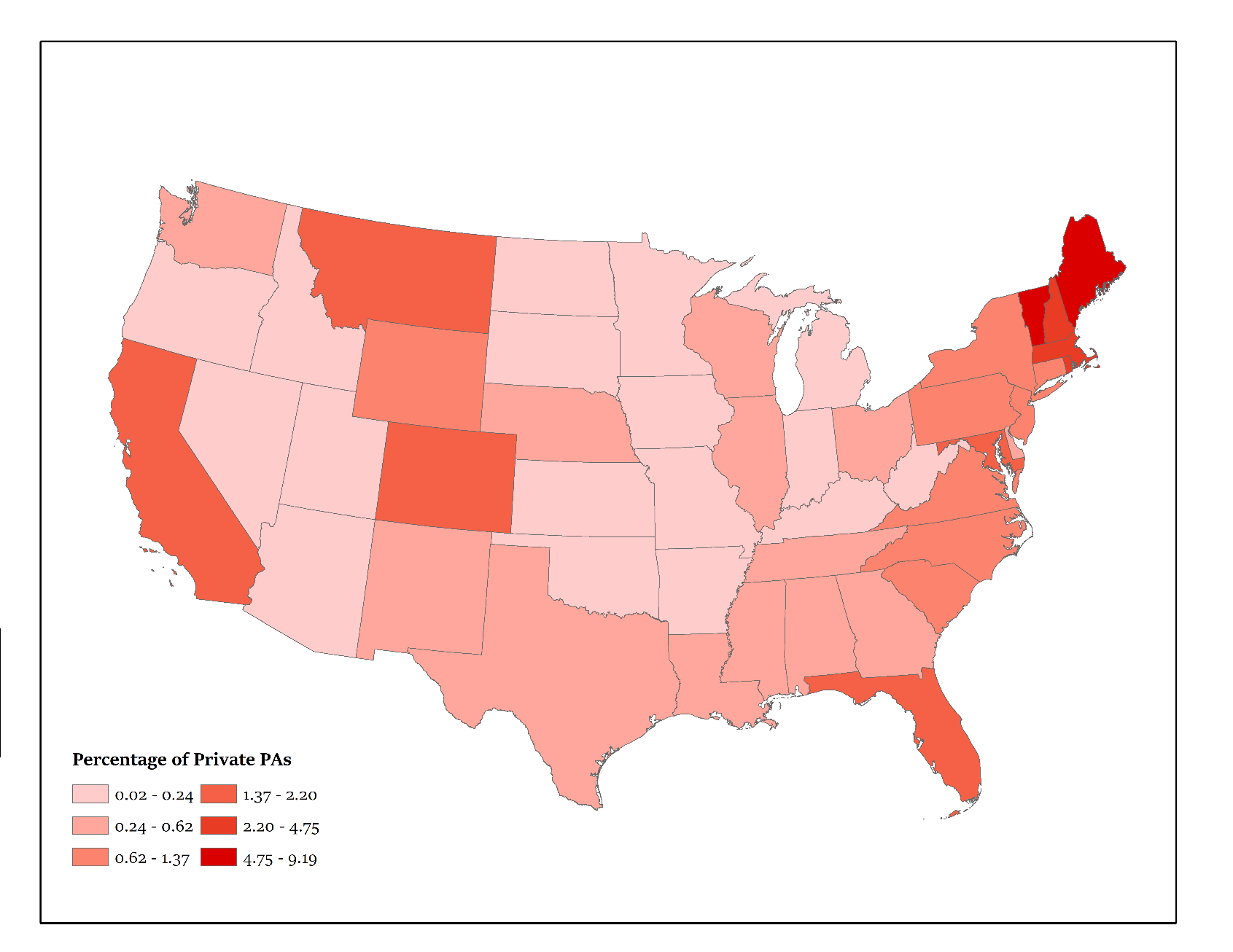

Supplement: S2 Fig — The average, by state, is 1.1% (range: 0.0% (Nevada) to 9.2% (Maine)). (TIF) [file pone.0228946.s006.tif]

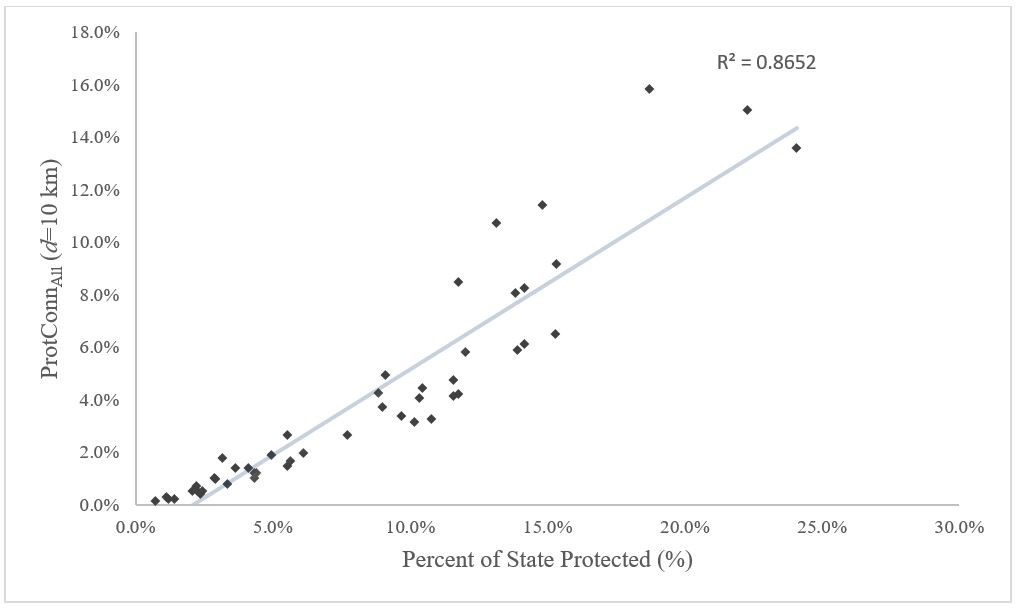

Supplement: S3 Fig — (JPG) [file pone.0228946.s007.JPG]

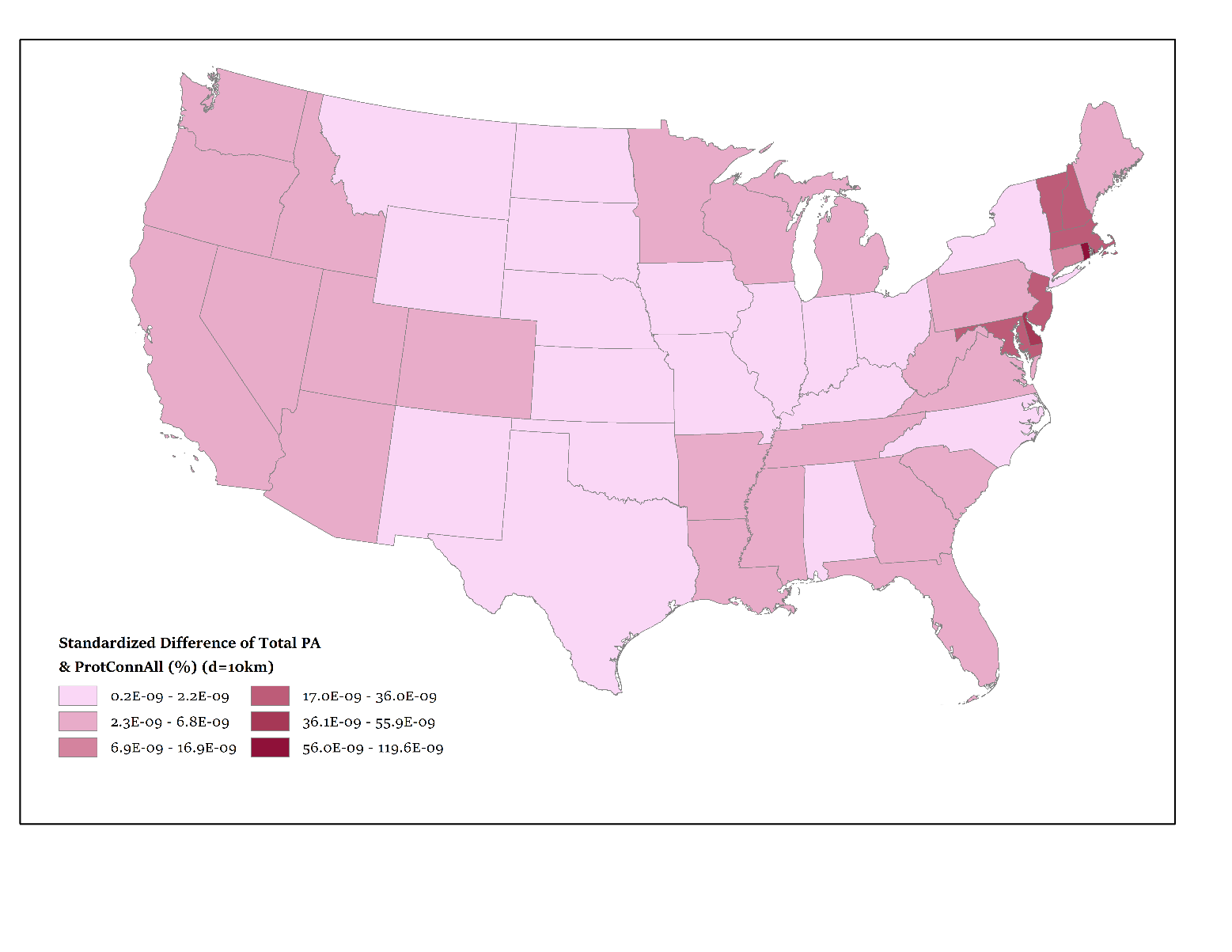

Supplement: S4 Fig — (TIF) [file pone.0228946.s008.tif]

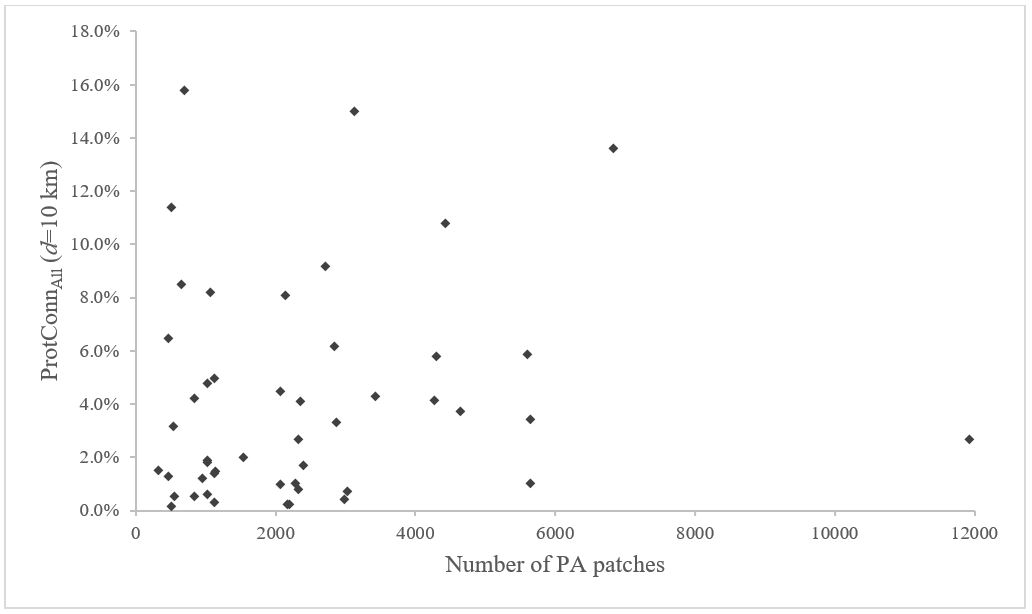

Supplement: S5 Fig — (JPG) [file pone.0228946.s009.JPG]

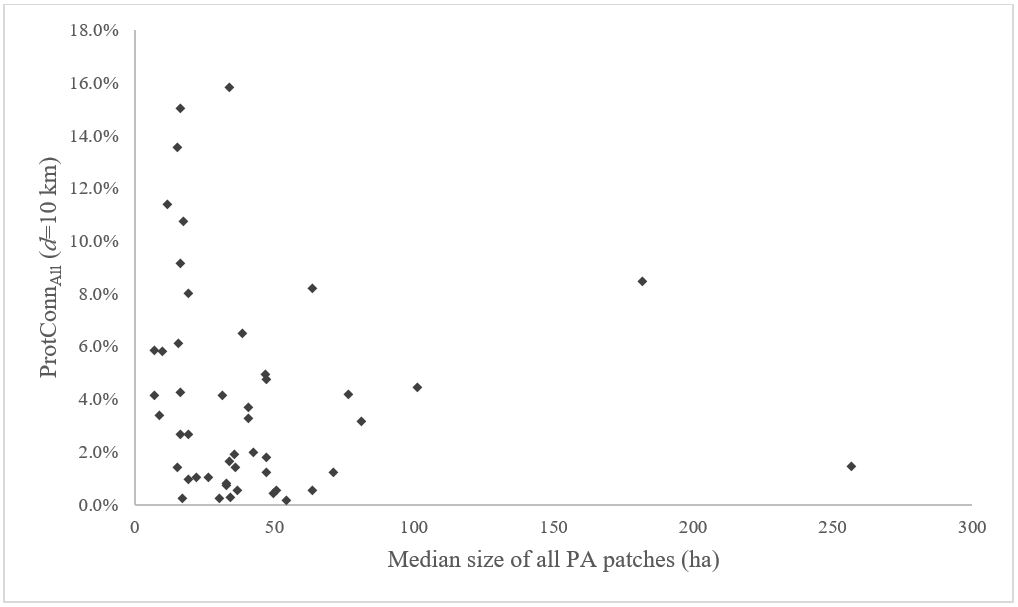

Supplement: S6 Fig — (JPG) [file pone.0228946.s010.JPG]

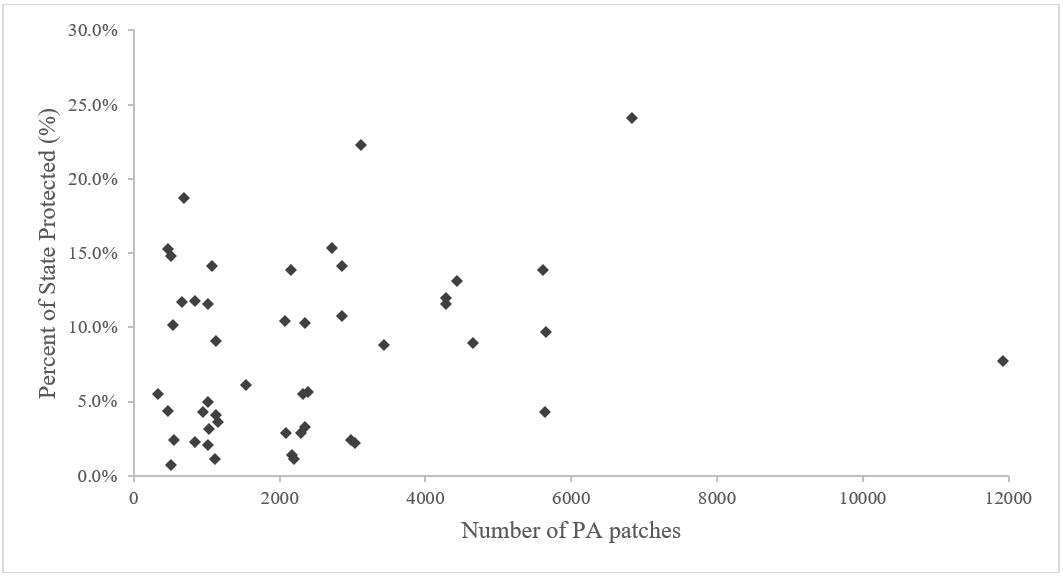

Supplement: S7 Fig — (JPG) [file pone.0228946.s011.JPG]

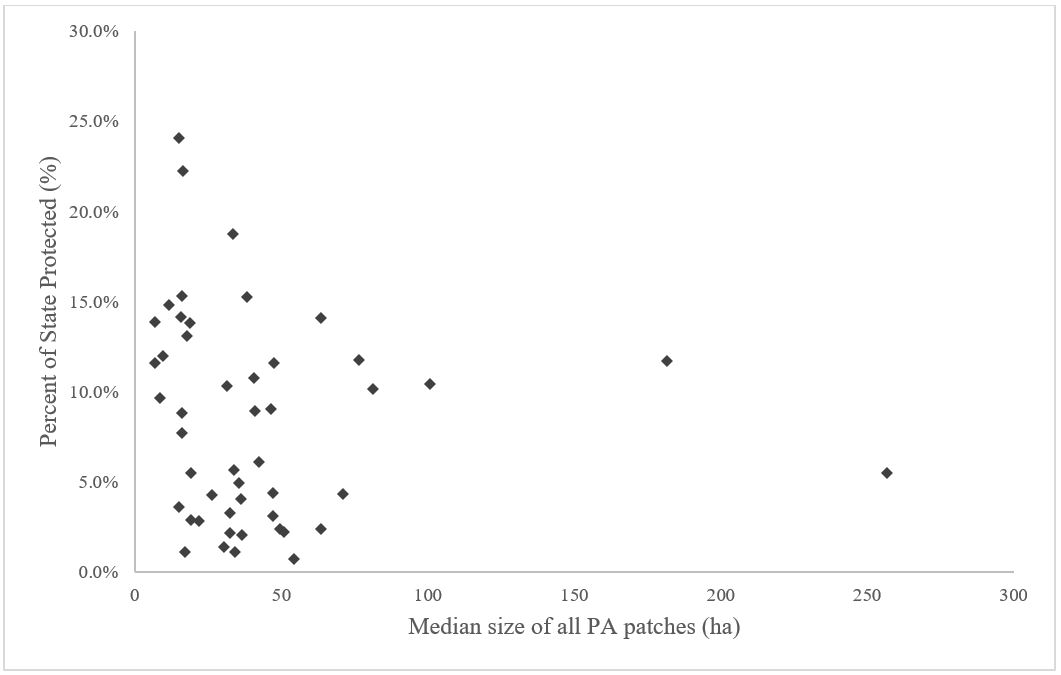

Supplement: S8 Fig — (JPG) [file pone.0228946.s012.JPG]

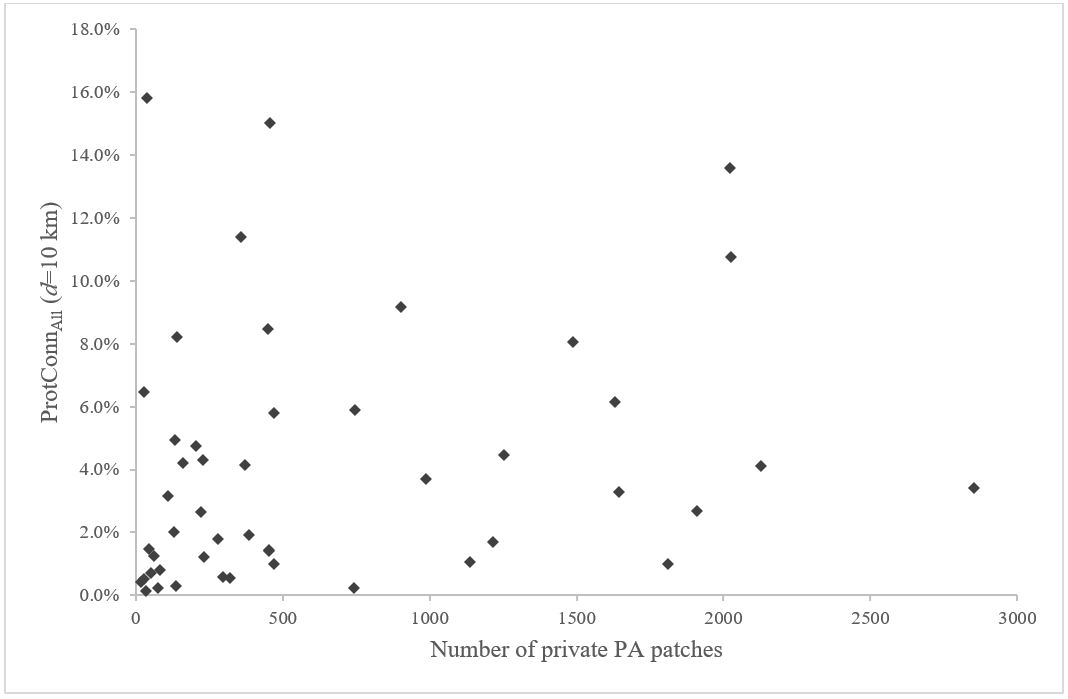

Supplement: S9 Fig — (JPG) [file pone.0228946.s013.JPG]

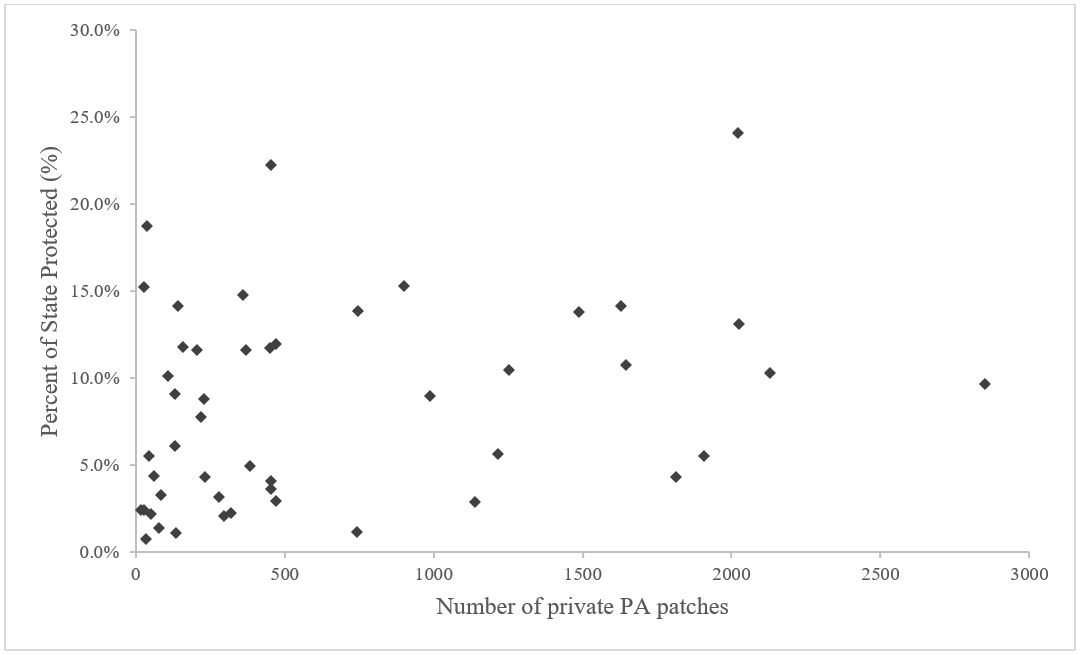

Supplement: S10 Fig — (JPG) [file pone.0228946.s014.JPG]

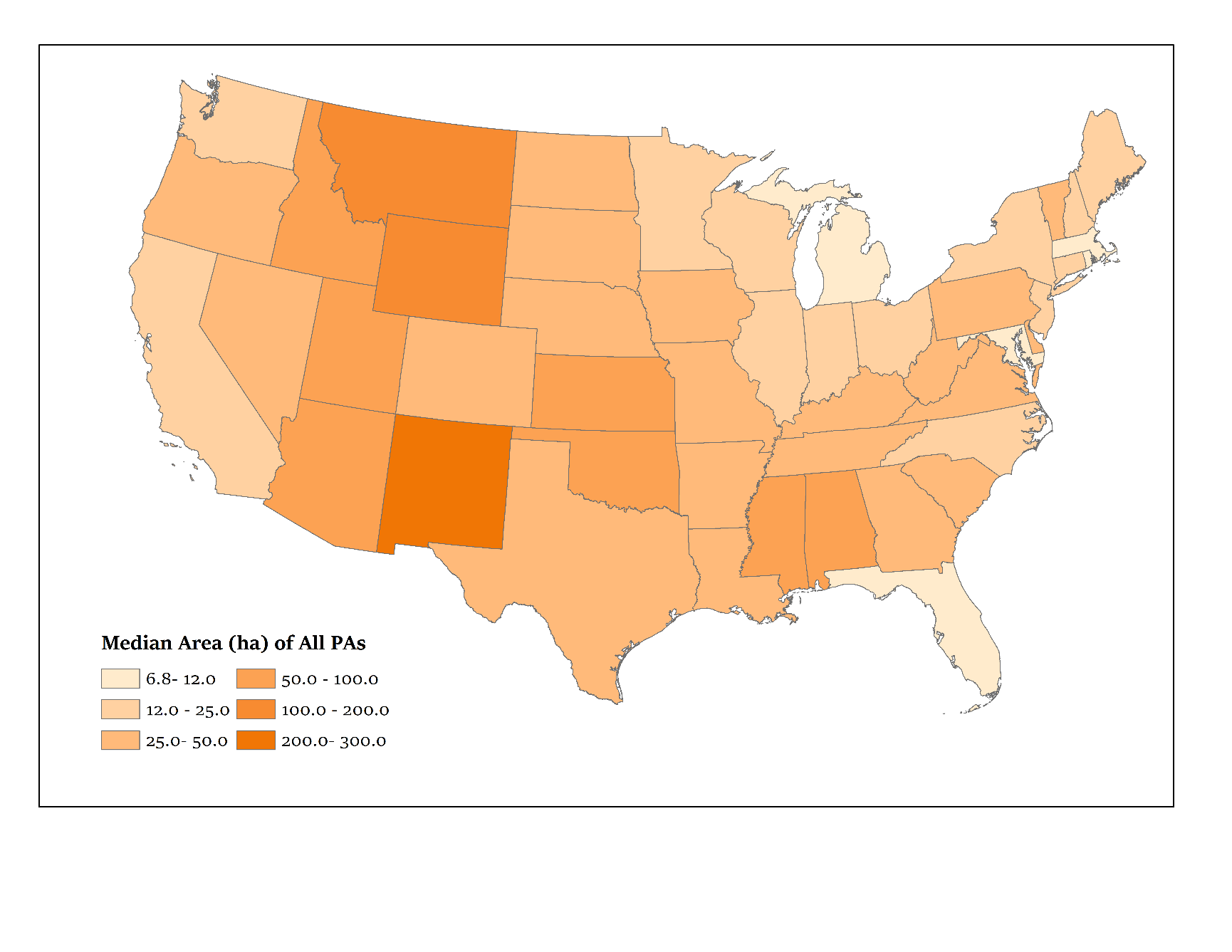

Supplement: S11 Fig — (TIF) [file pone.0228946.s015.tif]

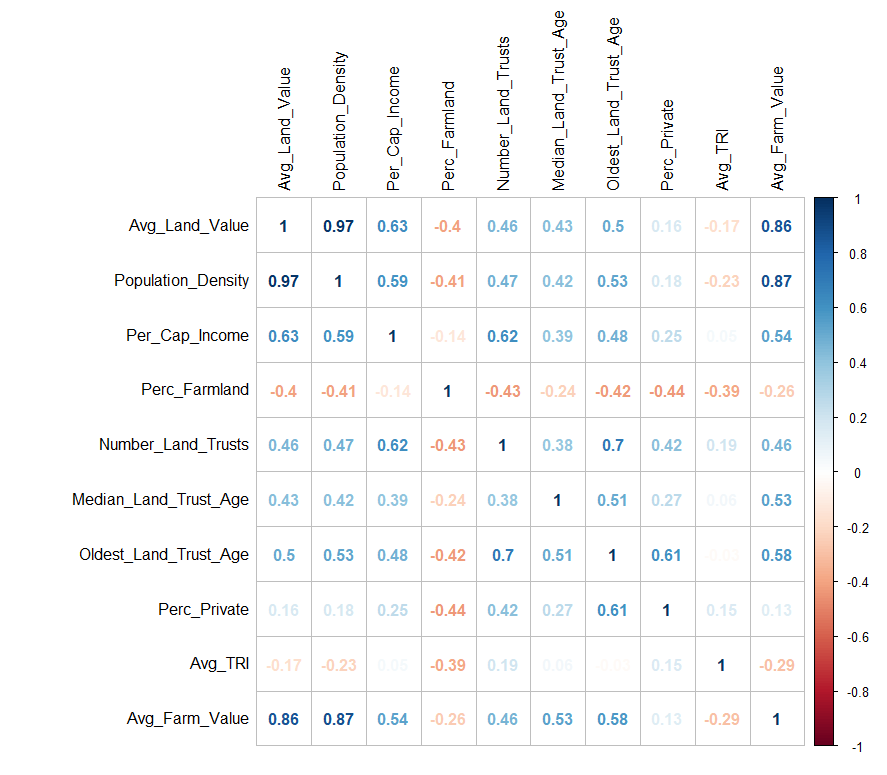

Supplement: S12 Fig — Predictors that were removed after testing for collinearity (r > 0.70) are: average land value, average farm value, the number of land trusts, and the age of the oldest land trust. (TIF) [file pone.0228946.s016.tif]

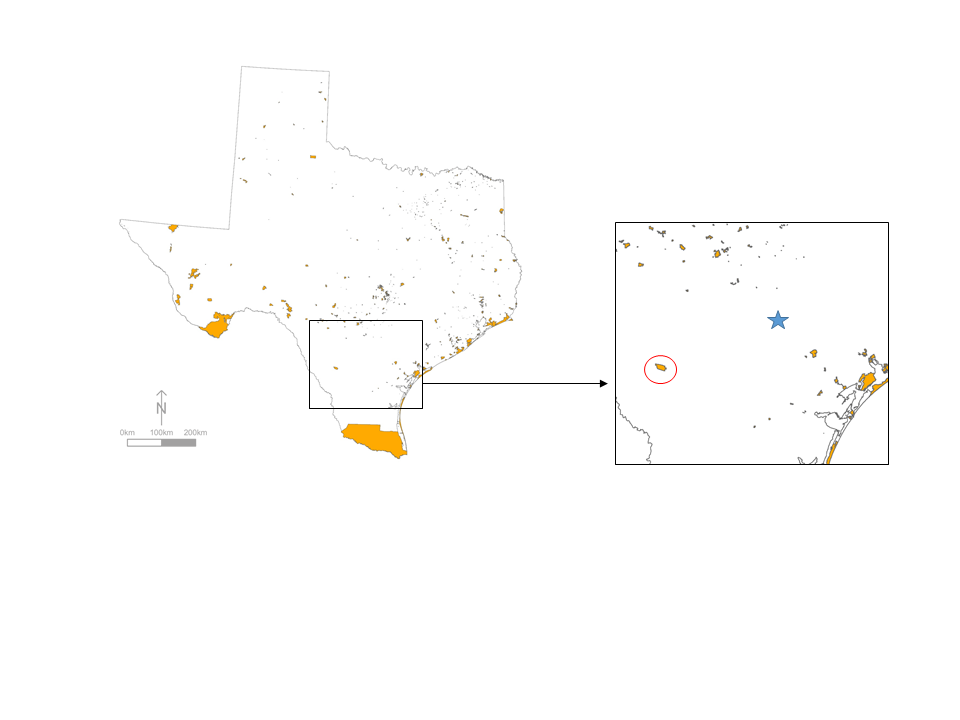

Supplement: S13 Fig — For illustrative purposes only, as the ulterior motive for designating this area is unknown, the alternative placement would have served as a stepping stone between patches of protected areas. (TIF) [file pone.0228946.s017.tif]
